# Spermine Alleviates Acute Liver Injury by Inhibiting Liver-Resident Macrophage Pro-Inflammatory Response Through ATG5-Dependent Autophagy

**DOI:** 10.3389/fimmu.2018.00948

**Published:** 2018-05-02

**Authors:** Shun Zhou, Jian Gu, Rui Liu, Song Wei, Qi Wang, Hongbing Shen, Yifan Dai, Haoming Zhou, Feng Zhang, Ling Lu

**Affiliations:** ^1^Liver Transplantation Center, First Affiliated Hospital, Nanjing Medical University, Nanjing, China; ^2^Department of General Surgery, Second Affiliated Hospital, Nanjing Medical University, Nanjing, China; ^3^Jiangsu Key Laboratory of Cancer Biomarkers, Prevention and Treatment, Collaborative Innovation Center for Cancer Personalized Medicine, Nanjing Medical University, Nanjing, China; ^4^Jiangsu Key Laboratory of Xenotransplantation, Nanjing Medical University, Nanjing, China

**Keywords:** liver injury, thioacetamide, spermine, Kupffer cell, polarization, autophagy, ATG5

## Abstract

Liver-resident macrophages (Kupffer cells, KCs) and autophagy play critical roles in the pathogenesis of toxin-induced liver injury. Recent evidence indicates that autophagy can regulate macrophage M1/M2 polarization under different inflammatory conditions. Polyamines, including putrescine, spermidine, and spermine (SPM), are polycations with anti-oxidative, anti-aging, and cell autophagy induction properties. This study aimed to determine the mechanisms by which SPM protects against thioacetamide (TAA)-induced acute liver injury in a mouse model. Pretreatment with SPM significantly alleviated liver injury and reduced intrahepatic inflammation in TAA-induced liver injury compared to controls. SPM markedly inhibited M1 polarization, but promoted M2 polarization of KCs obtained from TAA-exposed livers, as evidenced by decreased *IL-1*β and *iNOS* gene induction but increased *Arg-1* and *Mrc-1* gene induction accompanied by decreased STAT1 activation and increased STAT6 activation. Furthermore, pretreatment with SPM enhanced autophagy, as revealed by increased LC3B-II levels, decreased p62 protein expression, and increased ATG5 protein expression in TAA-treated KCs. Knockdown of ATG5 in SPM-pretreated KCs by siRNA resulted in a significant increase in pro-inflammatory TNF-α and IL-6 secretion and decreased anti-inflammatory IL-10 secretion after TAA treatment, while no significant changes were observed in cytokine production in the TAA treatment alone. Additionally, the effect of SPM on regulation of KC M1/M2 polarization was abolished by ATG5 knockdown in TAA-exposed KCs. Finally, *in vivo* ATG5 knockdown in KCs abrogated the protective effect of SPM against TAA-induced acute liver injury. Our results indicate that SPM-mediated autophagy inhibits M1 polarization, while promoting M2 polarization of KCs in TAA-treated livers *via* upregulation of ATG5 expression, leading to attenuated liver injury. This study provides a novel target for the prevention of acute liver injury.

## Introduction

The liver plays a crucial role in metabolic elimination of most of the currently used drugs and many other foreign compounds, thereby making it one of the most viable target organs for toxicity (1). Thioacetamide (TAA), a classic hepatotoxin that is also a potent carcinogen and mutagen, induces oxidative stress and sterile inflammation, resulting in acute and chronic liver injury (2, 3). Despite an increased understanding of the pathophysiology of toxin-induced liver injury, the precise mechanism of hepatotoxicity remains unclear.

Liver sterile inflammation caused by the innate immune response of liver-resident macrophages (Kupffer cells, KCs) plays a major role in the pathogenesis of toxin-induced liver injury (4). KCs are resident and non-migratory phagocytes serving as sentinels for liver homeostasis (5). During hepatic sterile inflammation, activated KCs release inflammatory cytokines and chemokines and attract other inflammatory cells to foci of injured tissue leading to the inflammatory signal amplification (6).

Macrophages have different functional states with a pro-inflammatory M1 type and an anti-inflammatory M2 type. The M1 macrophage phenotype is controlled by STAT1 and IRF5, whereas STAT6, IRF4, and PPARγ regulate M2 macrophage polarization (7). Autophagy is a homeostatic degradative process that removes damaged organelles and turns over cytoplasmic constituents through lysosomal compartments in eukaryotic cells (8). Recent studies have demonstrated the regulatory role of autophagy in macrophages. In obesity-induced hepatic steatosis, impaired macrophage autophagy increases liver inflammation and injury from lipopolysaccharide (LPS) by promoting pro-inflammatory M1 macrophage polarization (9). An inverse relationship between autophagy induction and maturation of NLR family pyrin domain containing 3 (NLRP3) inflammasomes in macrophages suggests another mechanism by which autophagy may influence macrophage activation (10).

Polyamines, such as putrescine, spermidine, and spermine (SPM), are aliphatic cations that interact with nucleic acids and proteins, functioning as modulators for cell growth, cell differentiation, and synthesis of DNA, RNA, and proteins (11). Polyamines are catabolized by back-conversion through acetylation and oxidation mediated by spermidine/spermine *N*1-acetyltransferase (SSAT) and *N*′-acetylpolyamine oxidase (APAO), respectively, or oxidation of SPM by SPM oxidase (SMO) (12). Dysregulation of polyamine metabolism is frequently associated with cancer and other hyperproliferative diseases (13). Several previous studies have demonstrated that polyamines protect against liver injury induced by drugs such as carbon tetrachloride and ethanol (14, 15). A recent study found that oral administration of polyamines ameliorates liver ischemia/reperfusion injury and promotes liver regeneration in rats. However, the mechanism by which polyamines protect against liver injury remains obscure.

Evidence is accumulating that polyamines are novel autophagy inducers and longevity elixirs (16). SPM increases autophagy by directly binding to p53 and p21 promoters (17). Furthermore, SPM ameliorates ischemia/reperfusion injury in cardiomyocytes *via* regulation of autophagy (18). Polyamines also function as anti-inflammatory factors through inhibition of pro-inflammatory cytokines and chemokine synthesis (19–21). In macrophages, basal polyamine levels are low and increase upon IL-4 or IL-13 stimulation, indicating that polyamines may regulate macrophage function (22).

Given the findings mentioned above, we explored the role of SPM in regulating liver injury and inflammation/immune activation in a TAA-treated mouse model with a focus on KC autophagy and polarization.

## Materials and Methods

### Animals

Eight-week-old male mice (C57BL/6J; the Laboratory Animal Resources Center of Nanjing Medical University, China) were house in constant environmental conditions under a standard rodent diet and water. All animals received humane care and all animal procedures met the relevant legal and ethical requirements according to a protocol (number NMU08-092) approved by the Institutional Animal Care and Use Committee of Nanjing Medical University.

### TAA-Induced Acute Liver Injury

After 1-week of acclimatization, the mice were intraperitoneally injected with a dose of 500 mg/kg TAA (Sigma, Saint Louis, MO, USA) dissolved in PBS. Normal control mice received the same volume of PBS *via* intraperitoneal injection. For SPM (Sigma, Saint Louis, MO, USA) supplementation studies, separate group of mice received intraperitoneal administration of SPM dissolved in PBS (10 mg/kg, twice daily, for 3 days) prior to TAA administration. The mice were divided into four groups: CON group, SPM group, TAA group, and TAA + SPM group (*n* = 6/group). The mice that received TAA administration alone were randomly sacrificed after 0, 6, 12, 24, and 48 h, six mice were sacrificed at each time point. Other groups were sacrificed at 24 h after TAA treatment. In some experiments, mice were pretreated with chloroquine (CQ, 60 mg/kg, i.p.) for 1 h prior to TAA administration. Serum and liver samples were collected. Tissues were stored continuously in liquid N_2_. Sections from the dissected livers were also fixed in 10% neutral buffered formalin for histological analysis.

### *In Vivo* ATG5 Knockdown

ATG5 siRNA (Santa Cruz, CA, USA) was premixed with mannose-conjugated polymers (Polyplus transfection, USA) at a ratio specified by the manufacture and was administered by tail vein injection (siRNA 2 mg/kg) 4 h prior to the TAA administration as described previously (23).

### Biochemical Analysis of Serum

Blood samples were collected and centrifuged to obtain serum for analysis. We used an automated chemical analyzer (Olympus Company, Tokyo, Japan) to detect the hepatic serum levels of alanine aminotransferase (ALT).

### Histopathology

Liver tissues were collected and incubated in 4% paraformaldehyde for at least 24 h, then embedded in paraffin. Sections (4 µm thick) were stained with hematoxylin eosin and used to observe inflammation and tissue damage by light microscopy.

### Caspase-3 Activity Assay

Caspase-3 activity was determined by an assay kit (Calbiochem, La Jolla, CA, USA) as described previously (24). Caspase-3 activity was assessed by measuring the absorbance at a wavelength of 405 nm with a plate reader.

### TUNEL Staining

Paraffin sections of hepatic tissues (4 µm thickness) were deparaffinated in toluene and then dehydrated by a graded series of ethanol solutions. TUNEL staining of liver tissues was performed using a fluorescent detection kit according to the manufacturer’s instructions.

### Immunohistochemical Staining

Liver macrophages and neutrophils were detected using primary rat anti-mouse F4/80, CD11b, and Ly6G mAb, respectively (BD Biosciences, San Jose, CA, USA). The secondary, biotinylated goat anti-rat IgG (Vector, Burlingame, CA, USA) was incubated with immunoperoxidase (ABC Kit, Vector), according to the manufacturer’s instruction. Positive cells were counted blindly in 10 HPF/section.

### KC Isolation and Cell Culture

Mouse livers were perfused *in situ via* the portal vein with HBSS, followed by 0.27% collagenase IV (Sigma, Saint Louis, MO, USA). Perfused livers were dissected and teased through 70-µm cell strainers, followed by suspension in 40 ml of DMEM supplemented with 10% FBS, 10 mM HEPES, 2 mM GlutaMax, 100 U/ml penicillin, and 100 mg/ml streptomycin for 15 min at 37°C, then the non-adherent cells were removed. The adherent cells were used for further *ex vivo* experiments. KCs were cultured *in vitro* for 6 h and then cells or supernatants were collected for further analysis. For M2 polarization, KCs isolated from mice were treated with 10 ng/ml IL-4 (Sigma, Saint Louis, MO, USA).

### Primary Mouse Hepatocytes

Primary mouse hepatocytes were isolated by a two-stage collagenase perfusion method as described previously (25). Hepatocytes were treated with TAA at 70 µM for 6 h in the presence or absence of SPM pretreatment at 50 µM for 2 h. Viability of hepatocytes was quantified by the Cell Counting Kit-8 assay according to the manufacture’s protocols (Dojindo Molecular Technologies, Inc., USA).

### Immunofluorescence Staining

LC3B, iNOS, and CD206 in KCs were identified by immunofluorescence using rabbit anti-mouse LC3B mAb, anti-mouse iNOS mAb, and anti-mouse CD206 mAb (Cell Signaling Technology, MA, USA). After incubation with secondary goat anti-mouse Texas Red-conjugated IgG (Sigma, Saint Louis, MO, USA), the KCs were pre-mounted with VECTASHIELD medium with DAPI (Vector). Positive cells were counted blindly in 10 HPF/section (200×). The positive cells were expressed as a percentage of total cells.

### Western Blots

Cellular proteins were extracted with ice-cold lysis buffer [1% Triton X-100, 0.5% sodium deoxycholate, 0.1% SDS, 10% glycerol, 137 mM sodium chloride, 20 mM Tris (pH 7.4)]. Proteins (20 µg) were subjected to 10% SDS-PAGE and transferred to polyvinylidene difluoride nitrocellulose membrane. Abs against p62, LC3B, ATG5, p-STAT1, p-STAT6, and β-actin (Cell Signaling Technology, MA, USA) were used.

### Quantitative RT-PCR

Following the manufacturer’s instructions, total RNA was extracted from frozen liver tissue and cells using TRIZOL reagent (Invitrogen, Carlsbad, CA, USA) and was reverse-transcribed into cDNA using the Transcriptor First Strand cDNA Synthesis Kit (Roche, Indianapolis, IN, USA). Quantitative real-time PCR was performed using SYBR green (Roche, Indianapolis, IN, USA). The expression levels of target genes and the results were normalized against GAPDH expression.

### ELISA

Cytokine (TNF-α, IL-6, IL-10, MCP-1, and CXCL-10) levels in cell culture supernatants or serum were measured by Elisa Kit according to the manufacture’s protocols (eBioscience, San Diego, CA, USA).

### Statistical Analysis

Results are shown as the mean ± SEM. Multiple group comparisons were performed using one-way analysis of variance followed by Bonferroni’s *post hoc* test. All analyses were performed using Stata software (version 11.0). *P* values less than 0.05 (two-tailed) were considered statistically significant.

## Results

### SPM Attenuates TAA-Induced Acute Liver Injury

**Figure 1:**
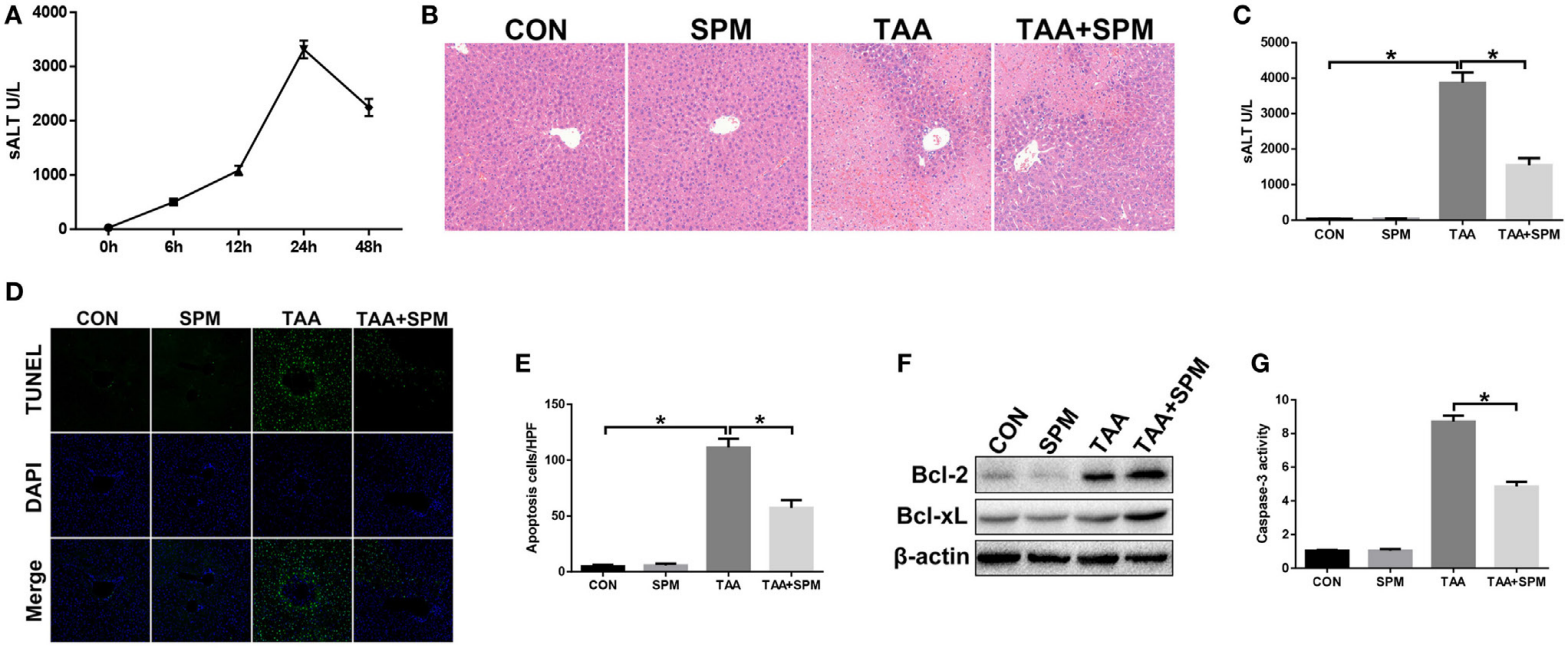
Spermine (SPM) pretreatment attenuates thioacetamide (TAA)-induced acute liver injury. Mice were subjected to SPM pretreatment and TAA administration as described in Section “Materials and Methods.” TAA-induced acute liver injury was evaluated by serum alanine aminotransferase (ALT) levels at different time points [**(A)**, *n* = 6/group]. Liver injury was evaluated in terms of liver histopathology [**(B)**, representative of six mice/group] and serum ALT [**(C)**, *n* = 6/group]. TUNEL staining of liver sections (original magnification 20×). DAPI was used for nuclear staining. Representative of six mice/group **(D)**. Ratio of TUNEL-positive cells in different experimental groups [**(E)**, *n* = 6/group]. Bcl-2, Bcl-xL, and β-actin protein levels were measured by Western blot. Representative of three experiments **(F)**. Cellular activity was determined by caspase-3 activity assay. Representative of three experiments **(G)** (*p < 0.05).

We first evaluated the severity of TAA-induced liver injury at different time points (0, 6, 12, and 24 h). As shown in Figure 1A, liver injury was most severe for 24 h after TAA administration, as evidenced by serum ALT levels. In light of this finding, all mice in this study were sacrificed 24 h after TAA administration.

Spermine treatment showed no toxicity in mice compared with the control (CON) group (Figures 1B–E). Compared with the TAA group, pretreatment with SPM significantly attenuated TAA-induced acute liver injury, as demonstrated by reduced liver necrosis (Figure 1B), lower serum ALT levels (Figure 1C), and less hepatocellular apoptosis (Figures 1D,E). Significantly higher levels of anti-apoptotic Bcl-2 and Bcl-xL and lower caspase-3 activity were observed in TAA + SPM-treated livers, compared to TAA treatment alone (Figures 1F,G). Thus, SPM treatment ameliorates TAA-induced acute liver injury.

### SPM Reduces Innate Immune and Inflammatory Responses in TAA-Exposed Livers

**Figure 2:**
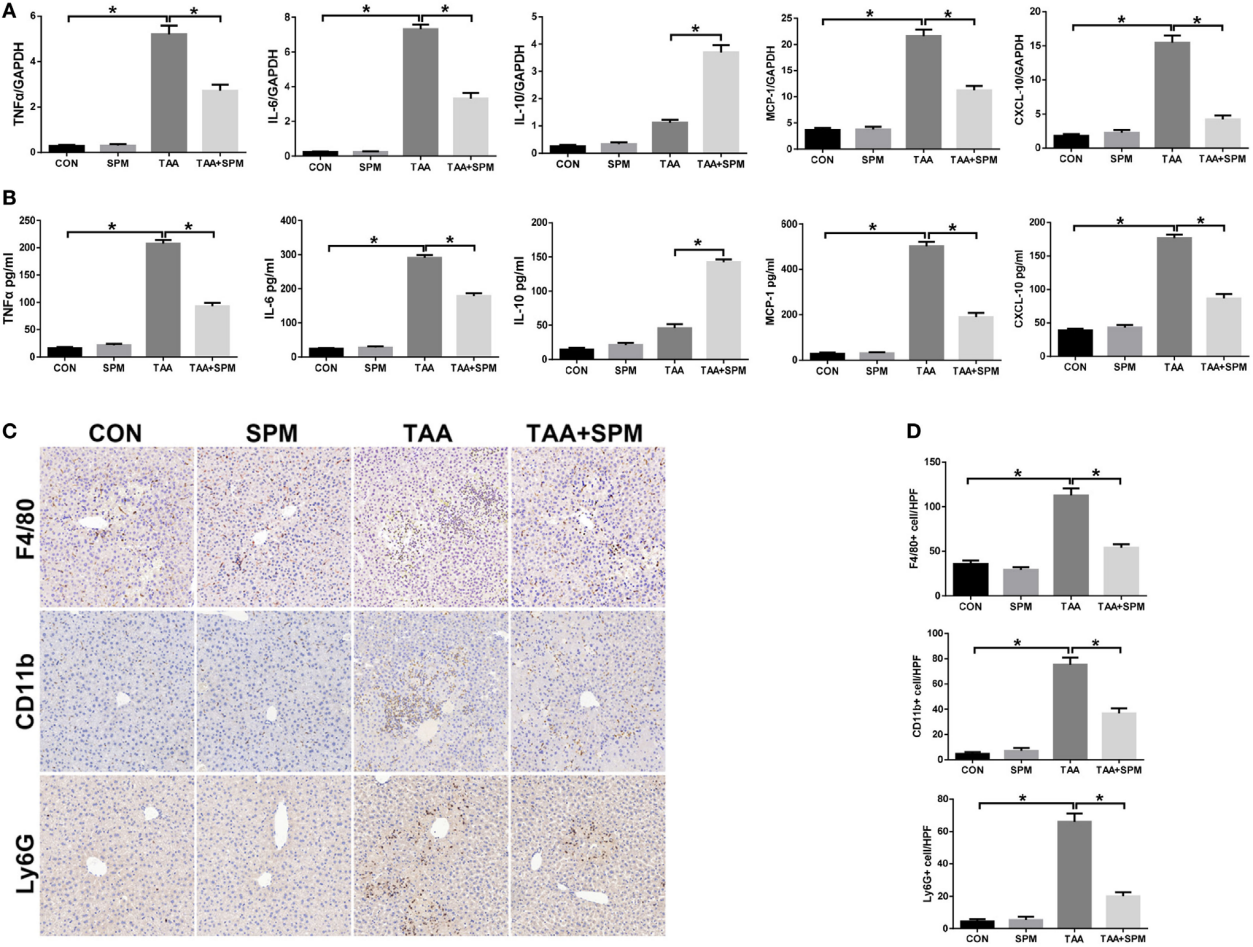
Spermine (SPM) pretreatment reduces innate immune and inflammatory responses in thioacetamide (TAA)-treated livers. Mice were subjected to SPM pretreatment and TAA administration as described in Section “Materials and Methods.” Inflammatory gene expression was measured in liver tissue by quantitative RT-PCR [**(A)**, *n* = 6/group]. Serum levels of inflammatory cytokines were measured by ELISA [**(B)**, *n* = 6/group]. F4/80^+^ macrophages, CD11b^+^ macrophages, and Ly6G^+^ neutrophils infiltration in liver was detected by immunohistochemical staining [**(C)**, *n* = 6/group]. Quantification of F4/80^+^ macrophages, CD11b^+^ macrophages, and Ly6G^+^ neutrophils per high power field (original magnification 20×). Representative of six mice/group **(D)** (*p < 0.05).

Innate immune inflammatory responses play a major role in TAA-induced acute liver injury (26). We examined KC relative cytokine and chemokine gene induction in different groups by qRT-PCR. As shown in Figure 2A, TAA treatment significantly increased *TNF-*α, *IL-6, MCP-1*, and *CXCL-10*, and slightly increased anti-inflammatory *IL-10* gene induction compared with the CON group. In contrast, TAA + SPM livers showed significantly lower *TNF-*α, *IL-6, MCP-1*, and *CXCL-10*, with higher anti-inflammatory *IL-10* gene induction compared to TAA treatment alone. In parallel, we observed that serum TNF-α, IL-6, MCP-1, and CXCL-10 levels were lower, while IL-10 levels were higher in the TAA + SPM group (Figure 2B). We next evaluated the effect of SPM on regulating peripheral macrophage and neutrophil infiltration by immunohistochemical staining. Interestingly, SPM significantly reduced the number of CD11b^+^ infiltrating macrophages and Ly6G^+^ neutrophils in TAA-challenged livers. Total intrahepatic macrophages (F4/80^+^) were reduced in TAA + SPM group as well (Figures 2C,D).

### SPM Regulates KC M1/M2 Polarization in Response to TAA Treatment

**Figure 3:**
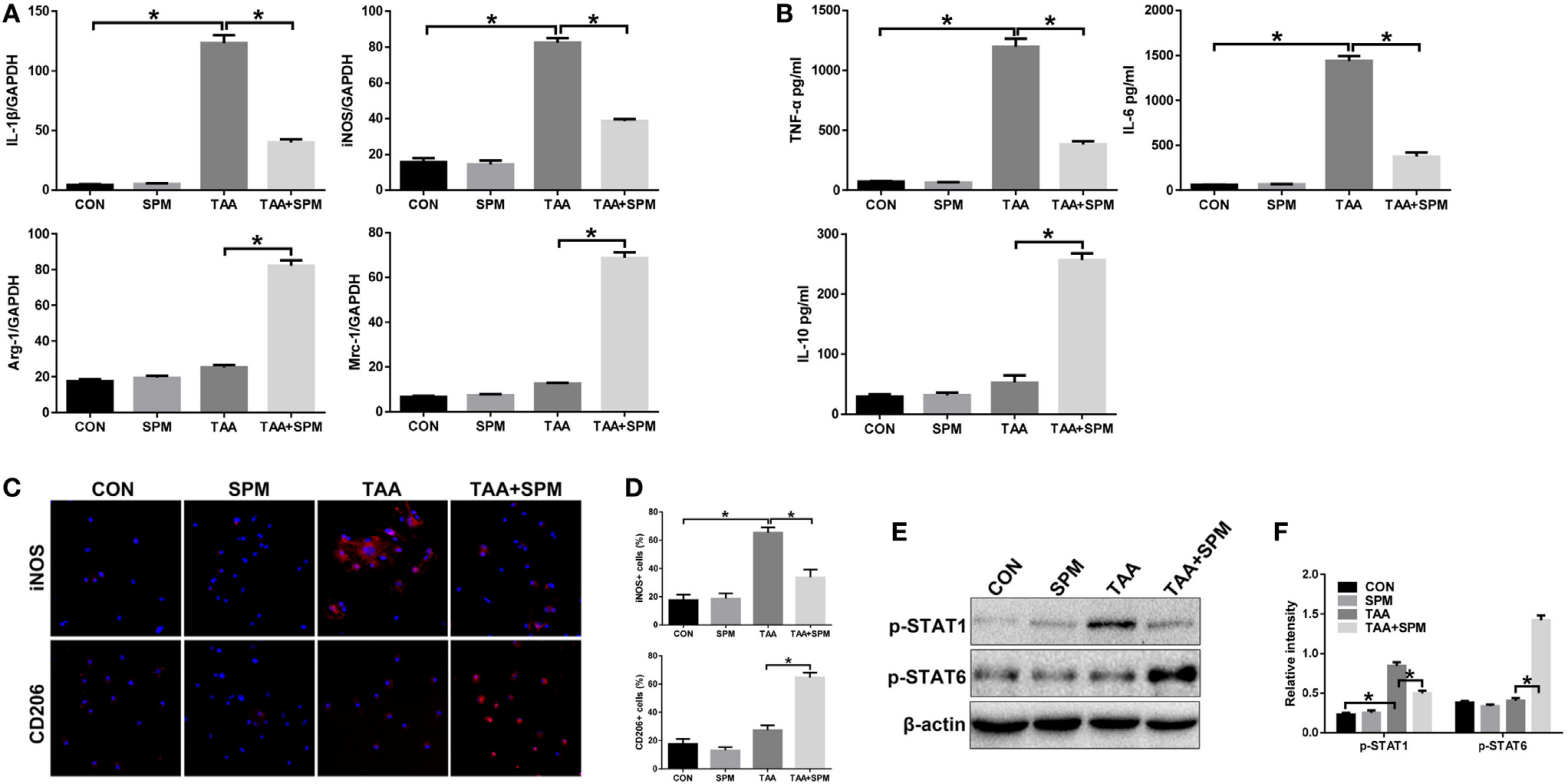
Spermine (SPM) regulates KC M1/M2 polarization in response to thioacetamide (TAA) treatment. Mice were subjected to SPM pretreatment and TAA administration as described in Section “Materials and Methods.” KCs were isolated from different experimental groups. M1 markers (*IL-1*β and *iNOS*) and M2 markers (*Arg-1* and *Mrc-1*) of gene induction were analyzed by quantitative RT-PCR. Representative of three experiments **(A)**. Isolated KCs from different experimental groups were cultured for 6 h, and TNF-α, IL-6, and IL-10 protein were measured in the culture supernatant by ELISA [**(B)**, *n* = 3/group]. Immunofluorescence staining of iNOS and CD206 in KCs (original magnification 20×). DAPI was used for nuclear staining [**(C)**, *n* = 3/group]. Ratio of iNOS^+^ and CD206^+^ cells in different experimental groups [**(D)**, *n* = 3/group]. Intracellular p-STAT1, (P)STAT6, and β-actin protein levels were measured by Western blot. Representative of three experiments **(E)**. Relative density ratios of target proteins in different experimental groups compared to the control group (CON) were calculated [**(F)**, *n* = 3/group] (*p < 0.05).

Macrophages can be broadly classified into M1 (classical) and M2 (alternative) subtypes based on function (27). We, therefore, determined the effect of SPM on regulating KC M1/M2 polarization. KCs isolated from each experimental group were plated and cultured *in vitro*. Indeed, after 6 h, KCs isolated from the TAA group exhibited higher induction of M1 markers (*IL-1*β and *iNOS*), but similar levels of M2 markers (*Arg-1* and *Mrc-1*) compared with the CON group (Figure 3A). We next analyzed levels of TNF-α, IL-6, and IL-10 protein in KC culture supernatant by ELISA. As shown in Figure 3B, higher levels of the pro-inflammatory cytokines TNF-α and IL-6 were secreted by TAA-treated KCs; however, no significant change was observed in levels of the anti-inflammatory cytokine IL-10 compared with CON KCs. The number of KCs positive for iNOS (M1 marker) significantly increased post TAA treatment compared to CON, but no significant influence in KCs positive for CD206 (M2 marker) was observed (Figures 3C,D). Furthermore, TAA treatment markedly increased activation of STAT1, but had no influence on activation of STAT6. These data indicate that KCs in TAA-induced livers preferentially polarized into the M1-like phenotype (Figures 3E,F).

Interestingly, pretreatment with SPM not only inhibited TAA-induced KC M1 polarization, but also promoted KC M2 polarization, as evidenced by lower levels of *IL-1*β and *iNOS* with higher levels of *Arg-1* and *Mrc-1* gene induction and lower levels of TNF-α and IL-6 with higher levels IL-10 in KC culture supernatant (Figures 3A,B). Decreased number of KCs positive for iNOS (M1 marker) and increased number of KCs positive for CD206 (M2 marker) in TAA + SPM group evaluated by immunofluorescence staining further confirmed the effect of SPM in inhibiting M1 polarization and promoting KC M2 polarization in response to TAA treatment (Figures 3C,D). Similar effects were found regarding STAT1 and STAT6 activation as detected by Western blot (Figures 3E,F). In addition, we detected STAT1 and STAT6 activation upon IL-4 stimulation *in vitro* by Western blot (Figure S1 in Supplementary Material). SPM treatment significantly increased STAT6 activation in KCs post IL-4 stimulation, which further confirmed that SPM promoted KC M2 polarization.

### SPM Induces KC Autophagy After TAA Treatment

**Figure 4:**
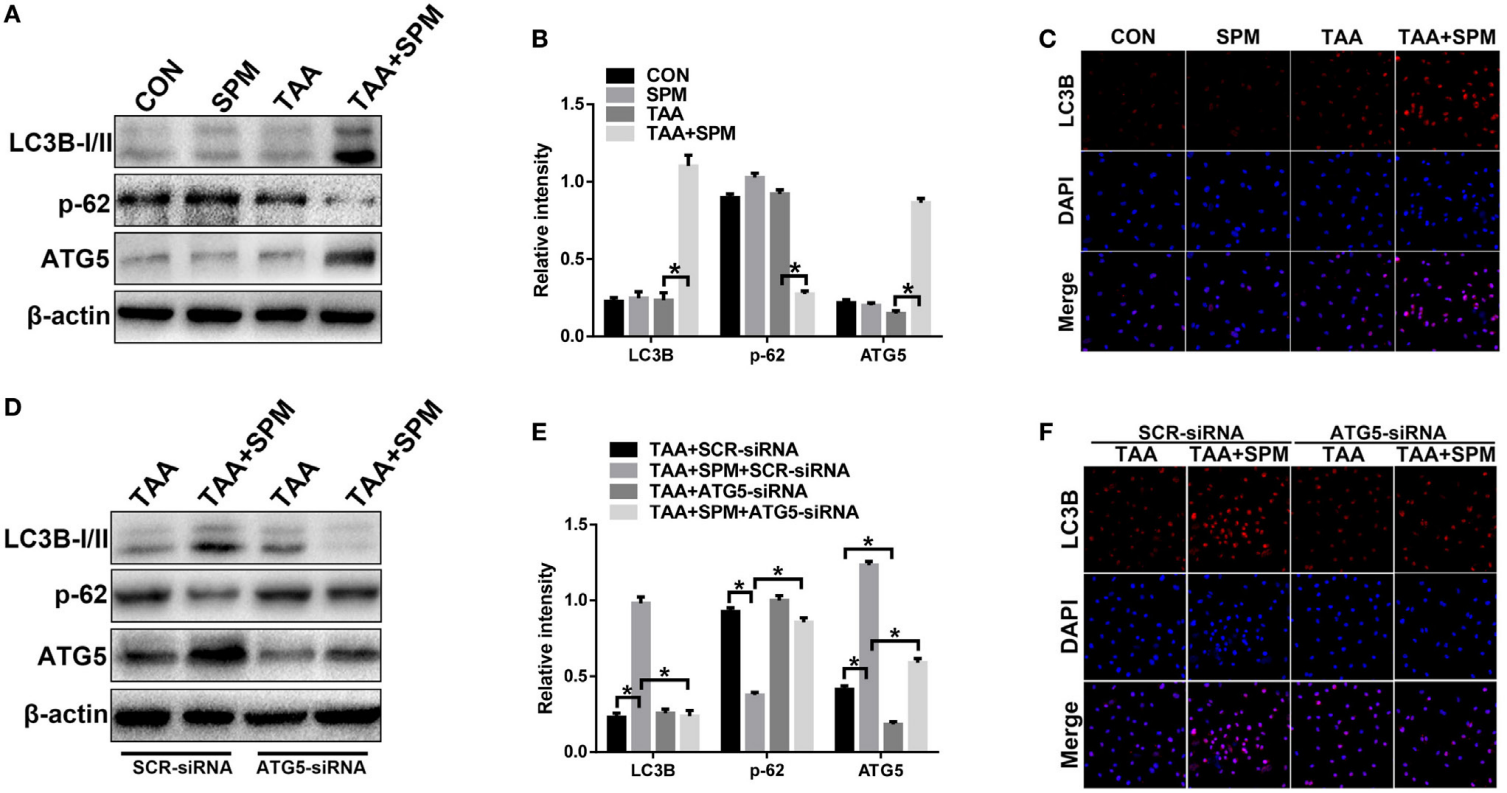
Spermine (SPM) induces KC autophagy after thioacetamide (TAA) treatment. Mice were subjected to SPM pretreatment and TAA administration as described in Section “Materials and Methods.” KCs were isolated from different experimental groups, and intracellular LC3B, p-62, ATG5, and β-actin protein levels were measured by Western blot. Representative of three experiments **(A)**. Relative density ratios of target proteins in different experimental groups compared to the control group (CON) were calculated [**(B)**, *n* = 3/group]. Immunofluorescence staining of LC3B in KCs (original magnification 20×). DAPI was used for nuclear staining [**(C)**, *n* = 6/group]. Both TAA and TAA + SPM mice were pretreated with ATG5 siRNA (ATG5-siRNA) or its scrambled control siRNA (SCR-siRNA) *in vivo* prior to TAA administration using mannose-conjugated polymers as described in “Materials and Methods.” KCs were isolated from different experimental groups and the intracellular LC3B, p-62, ATG5, and β-actin protein levels were measured by Western blot. Representative of three experiments **(D)**. Relative density ratios of target proteins in different experimental groups compared to the control group (CON-SCR-siRNA) were calculated [**(E)**, *n* = 3/group]. Immunofluorescence staining of LC3B in KCs (original magnification 20×). DAPI was used for nuclear staining [**(F)**, *n* = 6/group] (*p < 0.05).

Growing evidence illustrates that SPM is related to the autophagy pathway and cell survival (12, 28). Based on these findings, we examined whether SPM affects KC autophagy. KCs were isolated from each experimental group, and autophagy markers were analyzed by Western blot. As shown in Figures 4A,B and LC3BII levels were increased and p62 levels were decreased in TAA + SPM KCs but not in TAA KCs. LC3B immunofluorescence staining further confirmed that SPM induces KC autophagy after TAA treatment as evidenced by increased fluorescence intensity (Figure 4C). Autophagic flux was further analyzed by using chloroquine (CQ) (29, 30). Indeed, SPM pretreatment increased autophagic flux in KCs in response to TAA treatment, as indicated by significantly higher levels of LC3B-II in the presence versus absence of CQ. Furthermore, SPM pretreatment decreased protein levels of p62 in KCs, but CQ treatment significantly reversed the effect of SPM in TAA + SPM + CQ KCs (Figure S2 in Supplementary Material).

Interestingly, ATG5 was significantly upregulated in TAA + SPM KCs compared to KCs with TAA treatment alone (Figure 4A). These phenomena were absent in KCs without TAA treatment, indicating that SPM enhances KC autophagy in response to TAA treatment *via* increasing ATG5 expression.

### SPM Regulates KC M1/M2 Polarization in Response to TAA Treatment in an Autophagy-Dependent Manner

To evaluate whether the effects of SPM on regulation of KC M1/M2 polarization were directly related to autophagy induction, we utilized mannose-conjugated polymers to deliver ATG5 siRNA (ATG5-siRNA) or its scrambled control siRNA (SCR-siRNA) *in vivo* prior to TAA administration in mice with or without SPM pretreatment. KCs were subsequently isolated from each treatment group. ATG5-siRNA significantly inhibited ATG5 expression in both TAA and TAA + SPM KCs (Figures 4D,E). Furthermore, ATG5 knockdown significantly inhibited SPM-mediated autophagy in KCs as demonstrated by significantly decreased LC3B-II expression and increased p62 expression compared with SCR-siRNA treated control groups, but these effects were not observed in TAA KCs (Figures 4D,E). Similar results were evidenced by LC3B immunofluorescence staining (Figure 4F).

**Figure 5:**
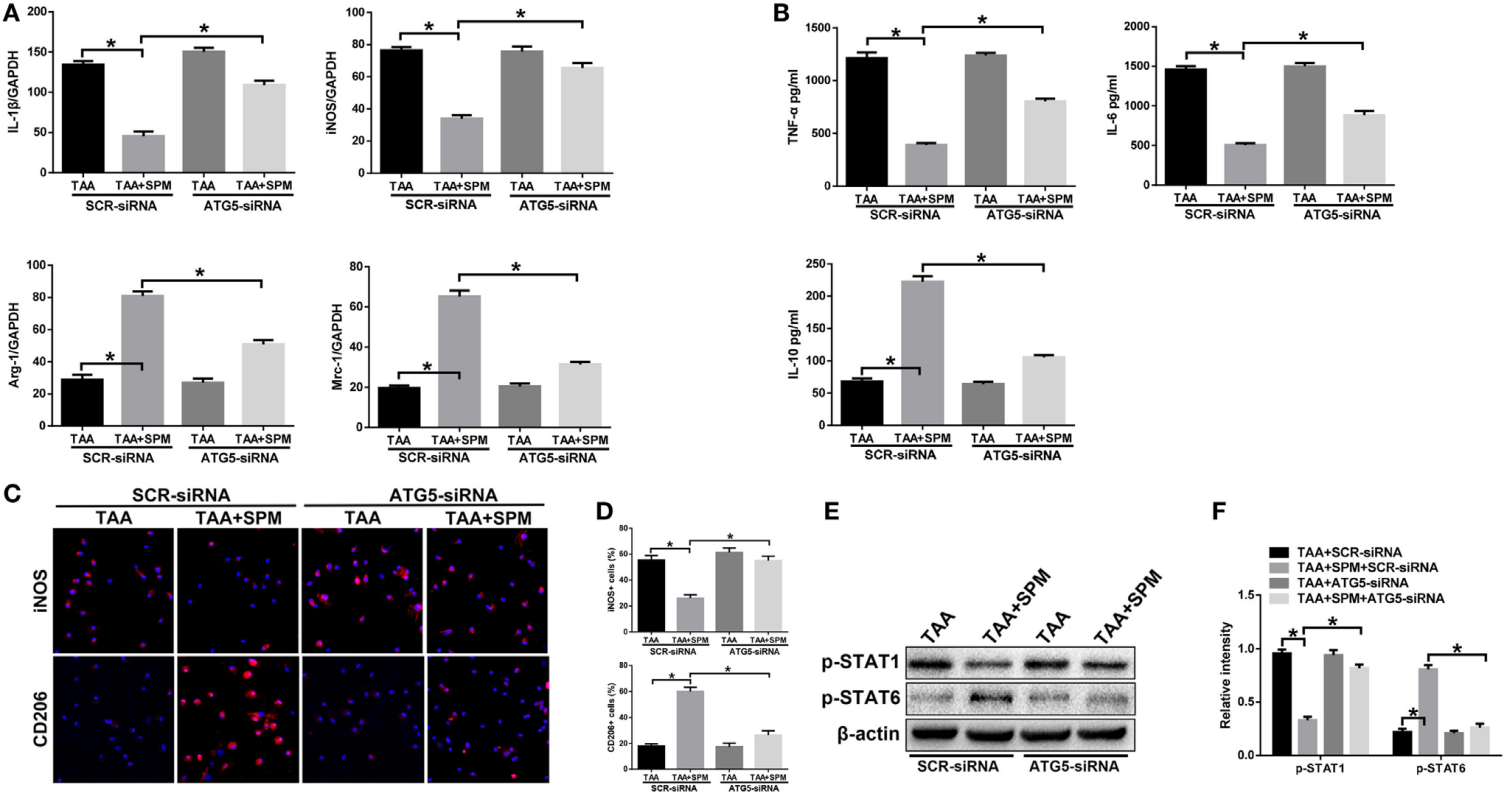
Spermine (SPM) regulates KC M1/M2 polarization in response to thioacetamide (TAA) treatment in an autophagy-dependent manner. Mice were subjected to SPM pretreatment and TAA administration as described in Section “Materials and Methods.” Both TAA and TAA + SPM mice were pretreated with ATG5 siRNA (ATG5-siRNA) or its scrambled control siRNA (SCR-siRNA) *in vivo* prior to TAA administration using mannose-conjugated polymers as described in Section “Materials and Methods.” KCs were isolated from different groups. M1 markers (*IL-1*β and *iNOS*) and M2 markers (*Arg-1* and *Mrc-1*) were measured by quantitative RT-PCR. Representative of three experiments **(A)**. Isolated KCs from different experimental groups were cultured for 6 h, and TNF-α, IL-6, and IL-10 protein were measured in the culture supernatant by ELISA [**(B)**, *n* = 3/group]. Immunofluorescence staining of iNOS and CD206 in KCs (original magnification 20×). DAPI was used for nuclear staining [**(C)**, *n* = 3/group]. Ratio of iNOS^+^ and CD206^+^ cells in different experimental groups [**(D)**, *n* = 3/group]. Intracellular p-STAT1, p-STAT6, and β-actin protein levels were measured by Western blot. Representative of three experiments **(E)**. Relative density ratios of target proteins in different experimental groups compared to the control group (CON-SCR-siRNA) were calculated [**(F)**, *n* = 3/group] (*p < 0.05).

Furthermore, ATG5 knockdown abolished SPM regulation of KC M1/M2 polarization in response to TAA treatment. Decreased induction of M1 markers (*IL-1*β, *iNOS*) and increased induction of M2 markers (*Arg-1, Mrc-1*) were abolished by ATG5 knockdown in TAA + SPM KCs compared to SCR-siRNA controls (Figure 5A). Furthermore, ATG5 knockdown increased secretion of pro-inflammatory cytokines TNF-α and IL-6, but decreased secretion of the anti-inflammatory cytokine IL-10 in TAA + SPM KCs (Figure 5B). iNOS and CD206 immunofluorescence staining results further confirmed that ATG5 knockdown abolished the regulatory role of SPM in KC M1/M2 polarization (Figures 5C,D). Similar effects were found regarding STAT1 and STAT6 activation as detected by Western blot (Figures 5E,F). In contrast, ATG5 knockdown had no remarkable effects in KCs treated with TAA in the absence of SPM.

These data demonstrate that SPM-mediated autophagy promotes M2 polarization and inhibits M1 polarization in KCs in response to TAA treatment.

### SPM Ameliorates TAA-Induced Acute Liver Injury by Inducing KC Autophagy

To investigate the role of autophagy in SPM attenuating TAA-induced liver injury *in vivo*, CQ was used to inhibit autophagy prior to TAA treatment. As shown in Figure S3 in Supplementary Material, the protective effect of SPM on TAA-induced liver injury is abrogated by CQ pretreatment, as evidenced by more severe liver damage and higher serum ALT level (TAA + SPM + CQ group versus TAA + SPM group). These results indicated the critical role of autophagy in protecting liver injury by regulating KC polarization.

**Figure 6:**
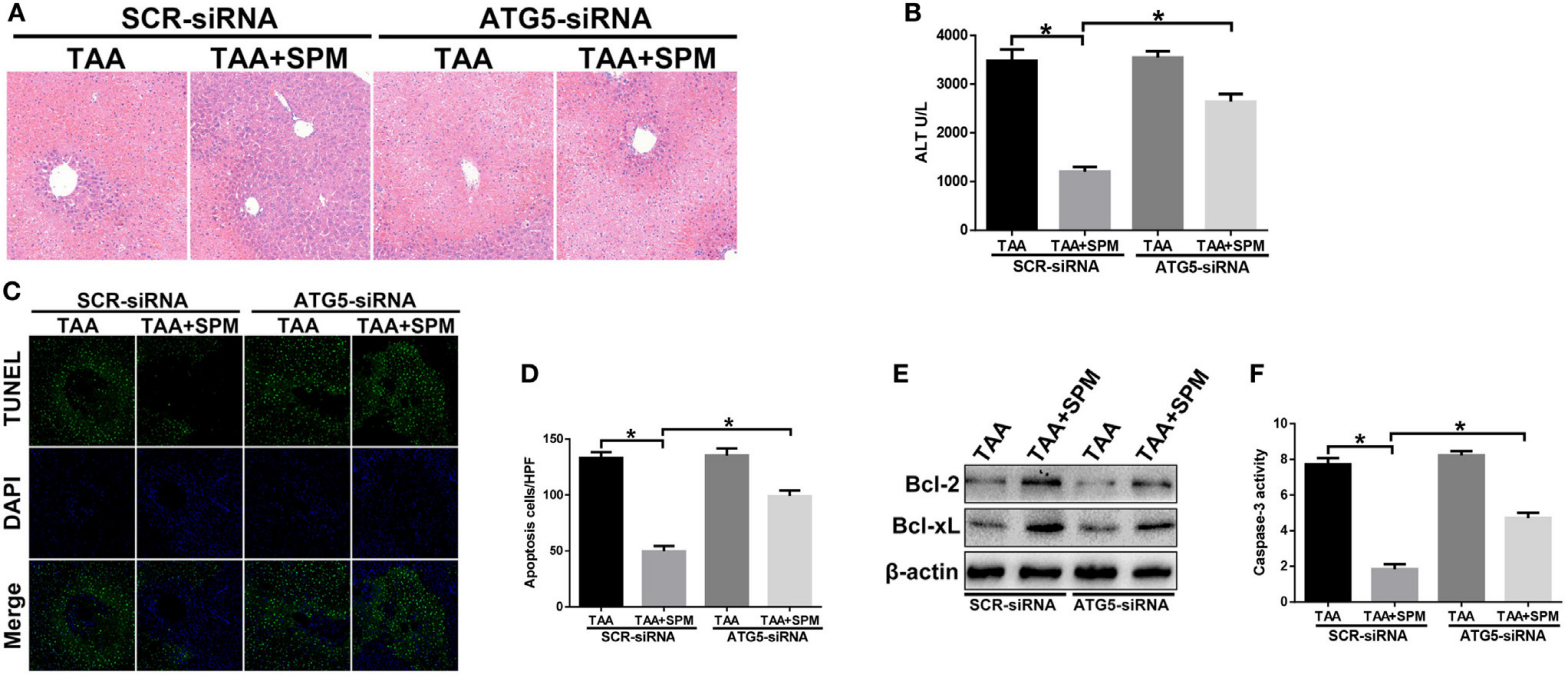
Spermine (SPM) attenuates thioacetamide (TAA)-induced acute liver injury by promoting KC autophagy. Mice were subjected to SPM pretreatment and TAA administration as described in Section “Materials and Methods.” Both TAA and TAA + SPM mice were pretreated with ATG5 siRNA (ATG5-siRNA) or its scrambled control siRNA (SCR-siRNA) *in vivo* prior to TAA administration using mannose-conjugated polymers as described in Section “Materials and Methods.” Liver injury was evaluated in terms of liver histopathology [**(A)**, representative of six mice/group] and serum alanine aminotransferase (ALT) levels [**(B)**, *n* = 6/group]. TUNEL staining of liver sections (original magnification 20×). DAPI was used for nuclear staining. Representative of six mice/group **(C)**. Ratios of TUNEL-positive cells in different experimental groups [**(D)**, *n* = 6/group]. Bcl-2, Bcl-xL, and β-actin protein levels were measured by Western blot. Representative of three experiments **(E)**. Cellular activity was determined by caspase-3 activity assay. Representative of three experiments **(F)** (*p < 0.05).

To further study the underlying mechanism of SPM in regulating KC autophagy, we specifically inhibited autophagy in macrophages *in vivo* using ATG5-siRNA prior to TAA administration. Interestingly, *in vivo* ATG5 knockdown abolished the protective role of SPM in TAA-induced liver injury, as evidenced by increased damage to liver architecture (Figure 6A), higher serum ALT levels (Figure 6B), and increased hepatocellular apoptosis (Figures 6C–F) compared to the SCR-siRNA control group. However, ATG5 knockdown had no significant effects in the group treated with TAA alone.

### SPM-Mediated KC Autophagy Inhibits Inflammatory/Immune Activation

**Figure 7:**
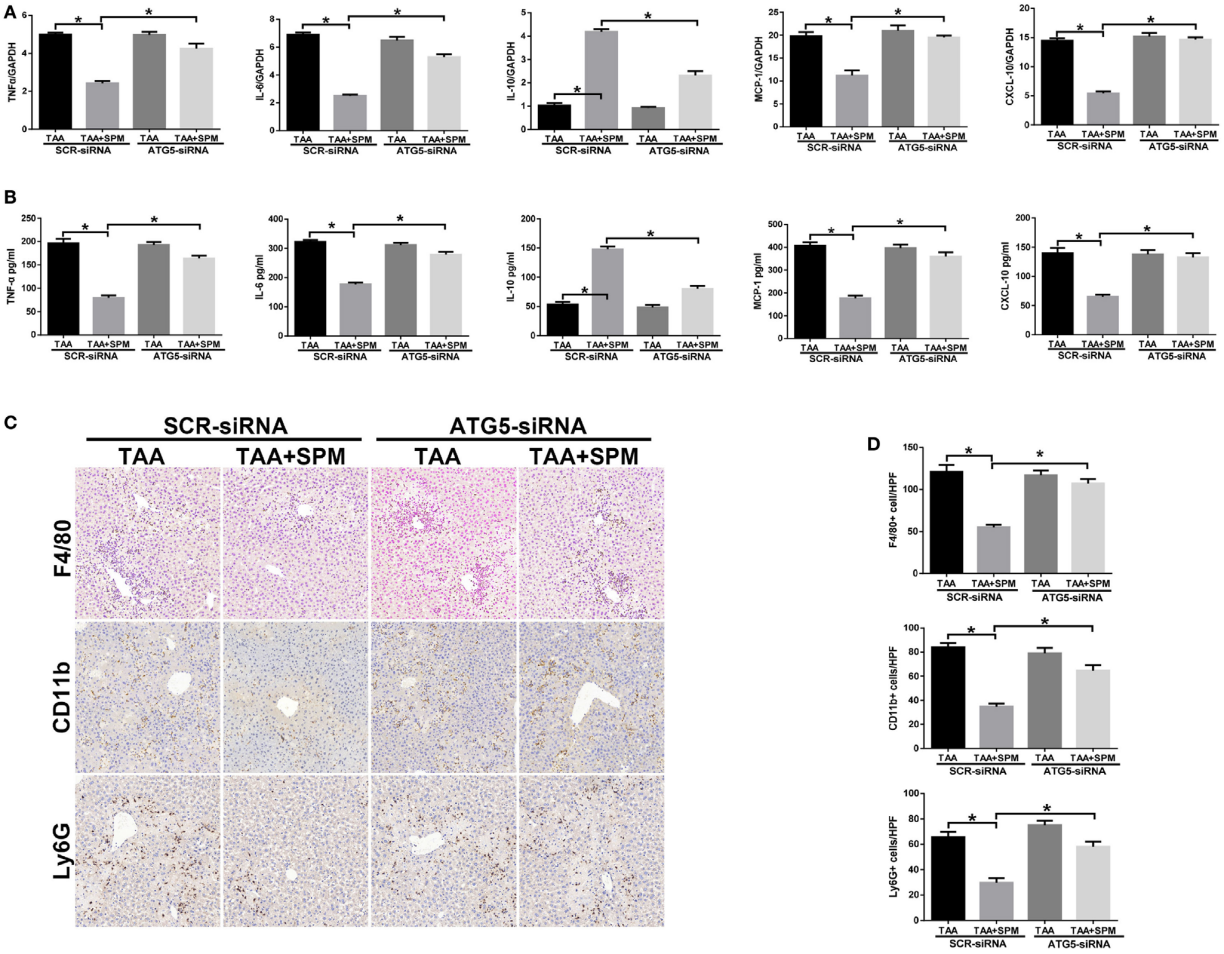
Spermine (SPM)-mediated KC autophagy inhibits inflammatory immune activation. Mice were subjected to SPM pretreatment and thioacetamide (TAA) administration as described in Section “Materials and Methods.” Both TAA and TAA + SPM mice were pretreated with ATG5 siRNA (ATG5-siRNA) or its scrambled control siRNA (SCR-siRNA) *in vivo* prior to TAA administration using mannose-conjugated polymers as described in Section “Materials and Methods.” Inflammatory gene expression in liver tissue was evaluated by quantitative RT-PCR [**(A)**, *n* = 6/group]. Serum levels of inflammatory cytokines were measured by ELISA [**(B)**, *n* = 6/group]. F4/80^+^ macrophages, CD11b^+^ macrophages, and Ly6G^+^ neutrophils infiltration in liver was detected by immunohistochemical staining [**(C)**, *n* = 6/group]. Quantification of F4/80^+^ macrophages, CD11b^+^ macrophages, and Ly6G^+^ neutrophils per high power field (original magnification 20×). Representative of six mice/group **(D)** (*p < 0.05).

Finally, we investigated the role of SPM-mediated KC autophagy in regulating innate immune activation in TAA-induced liver injury. We used ATG5-siRNA to suppress KC autophagy *in vivo*. As shown in Figure 7A, compared with SCR-siRNA controls, ATG5 knockdown in TAA + SPM livers significantly increased intrahepatic *TNF-*α, *IL-6, MCP-1*, and *CXCL-10* gene induction and decreased *IL-10* gene induction. Similar results were found in regarding serum levels of TNF-α, IL-6, MCP-1, CXCL-10, and IL-10 (Figure 7B). Furthermore, ATG5 knockdown significantly increased the number of CD11b^+^ infiltrating macrophages and Ly6G^+^ neutrophils in TAA + SPM group. Decreased total intrahepatic macrophages (F4/80^+^) were restored by ATG5 knockdown in TAA + SPM group as well (Figures 7C,D).

## Discussion

The results of this study reveal that pretreatment with SPM attenuate TAA-induced acute liver injury and decreases hepatocellular death through inhibition of KC innate immune inflammation. Although the anti-inflammatory and protective role of SPM in inflammatory diseases and organ damage has been well established (18, 21, 31–33), its role in TAA-induced acute liver injury remains to be elucidated. Our study identifies a novel mechanism by which SPM ameliorates innate immune inflammation in TAA-exposed livers. SPM induces KC autophagy *via* upregulation of ATG5 expression that in turn inhibits pro-inflammatory M1 polarization and promotes anti-inflammatory M2 polarization, thereby leading to attenuated intrahepatic inflammation and reduced hepatocellular injury in TAA-treated livers. To the best of our knowledge, this is the first study to demonstrate that SPM regulates KC polarization via induction of autophagy in the TAA-induced liver injury model.

Polyamines, especially SPM, are ubiquitous and essential in all living systems (34), possessing multi-functional properties that modulate several protein pathways and exert immune-modulatory properties. Indeed, it is already known that SPM concentrations significantly increase in tissues following injury, inflammation, and antigen stimulation, attributed to both its release from dying and injured cells and its stimulated biosynthesis (35). Interestingly, no significant difference was found in terms of hepatocellular survival and autophagy activation (LC3B-II levels) between the TAA and TAA + SPM groups in the present study, indicating that SPM had no direct effects on the liver parenchymal cells (Figure S4 in Supplementary Material).

Macrophages are the major cells that respond to liver injury and are responsible for triggering tissue inflammation, leading to neutrophil activation and infiltration (36). Szabo et al. first reported that polyamines inhibited overproduction of macrophage-induced inflammation and downregulated neutrophil locomotion (37). Consistent with previous studies, our *in vivo* data show that SPM markedly attenuates the inflammatory response in TAA-treated livers, evidenced by reduced mRNA levels of pro-inflammatory cytokines and decreased macrophage and neutrophil infiltration. These results beg the question of what mechanisms may confer SPM with the ability to regulate macrophage activity in TAA-induced acute liver injury.

Macrophages have different functional states, comprising a pro-inflammatory M1 type and an anti-inflammatory M2 type (38). M1 macrophages can be induced by LPS and IFN-γ, leading to release of pro-inflammatory cytokines and enzymes, such as IL-1β, TNF-α, and iNOS (39). In contrast, M2 macrophages express anti-inflammatory cytokines and enzymes, such as IL-10 and Arg-1 and can be induced by IL-4 and IL-13 stimulation (40). Zhang et al. indicated that LPS-induced production of pro-inflammatory cytokines was inhibited by SPM in human monocytes (20). Polyamines inhibit iNOS translation and l-arginine uptake as well, leading to decrease NO production and inhibition of antimicrobial activity (41). A recent study demonstrated that Arg-1-independent polyamine production stimulates expression of IL-4-induced M2 macrophage markers, while inhibiting LPS-induced expression of inflammatory genes (22). These studies all suggest that polyamines are intimately involved in macrophage polarization.

Kupffer cells are liver-resident macrophages that account for 20–35% of all non-parenchymal cells in the liver (42). KCs reside in the hepatic sinusoid and serve as a first line of defense against bacteria, microbial debris, and endotoxins in various liver diseases and injuries. The role of KC M1/M2 polarization in different liver diseases and injuries has been reported in many studies. A recent study showed that in a mouse liver ischemia/reperfusion model, hyperglycemia-induced CHOP over-activation inhibited KC M2 polarization, leading to excessive intrahepatic inflammation and exacerbation of liver IR injury (43). Nogo-B was shown to be permissive for M1 polarization in KCs by inhibiting ER stress, thereby accentuating liver injury in alcoholic liver disease in humans and mice (44). Indeed, in our present study, TAA treatment significantly promoted pro-inflammatory M1 polarization in KCs but had no influence on anti-inflammatory M2 polarization. We detected STAT1 and STAT6 activation by Western blot. Consistently, TAA treatment significantly activated STAT1 but had no effect on STAT6 activation. After activation, the KCs could not only secret pro-inflammatory cytokines, but also produce some chemokines, such as MCP-1 and CXCL-10, which could further attract some other immune cells (CD11b^+^ infiltrating macrophages and Ly6G^+^ neutrophils) into injured livers (45). A recent study indicated that activation of IL-1α in KCs plays a central role in the recruiting myeloid cells to sites of damage in a mouse mode of acetaminophen hepatotoxicity (46). In this study, both the numbers of infiltrating macrophages and neutrophils in the livers were decreased by SPM pretreatment. This is probably caused by decreased MCP-1 and CXCL-10 expression in KCs by SPM pretreatment in livers in response to TAA treatment. However, the direct role of SPM in regulating the infiltration of these cells was not analyzed in this study.

Furthermore, we found that SPM pretreatment markedly promoted M2 polarization and inhibited M1 polarization of KCs in TAA-treated groups but had no significant effect in control groups. This result may be related to polyamine metabolism states in different conditions. In physiological conditions, maintenance of polyamine catabolism homeostasis in animals is regulated by enzymatic functions, such as spermidine/spermine *N*1-acetyltransferase (SSAT), *N*1-APAO, SMO. In contrast, changes in polyamine catabolism in response to injury, inflammation, or inflammatory mediators have a wide range of effects whose eventual outcome is dependent on the stimuli, the specific cell type, and the cellular environment (47). *Helicobacter pylori* infection has been demonstrated to increase expression and activity of polyamine biosynthetic enzymes (33). Ischemia/reperfusion injury induces polyamine metabolism imbalance in rat myocardium, resulting in increased concentration of putrescine and decreased spermidine and SPM concentrations (48).

Autophagy is a conserved catabolic process in which cellular components are transported to and degraded in lysosomes (8). Emerging evidence has shown that autophagy is involved in regulation of macrophage M1/M2 polarization, but the effect remains controversial. *Brucella* infection suppresses both M1 and M2 macrophage polarization by inducing LC3B-related autophagy in patients (49). Isoprenaline promotes M2 macrophage polarization by downregulating autophagy and activating downstream ROS/ERK and mTOR signaling pathways (50). However, Kun Liu et al. reported that autophagy was required to suppress both M1 and promote M2 polarization in macrophages (9). It was recently reported that autophagy in macrophages plays an important role in acute liver injury. GAS6-AXL signaling-mediated autophagy induction in murine macrophages ameliorated hepatic inflammatory responses by inhibiting NLRP3 inflammasome activation in both LPS and carbon tetrachloride-induced models (51).

Spermine has been reported as a novel inducer of autophagy in a variety of studies (12, 16, 52); however, little is known about the involvement of SPM in autophagy induction of macrophages. We tested whether SPM regulated macrophage M1/M2 polarization *via* induction of autophagy. We first measured protein expression related to autophagy of LC3B and p62 in KCs isolated from different experimental groups by Western blot and LC3B immunofluorescence. Our results indicated that pretreatment with SPM significantly induces KC autophagy in TAA-treated groups but not in controls. Interestingly, ATG5 was markedly upregulated in TAA + SPM KCs, indicating that SPM-mediated KC autophagy occurs in an ATG5-dependent manner.

To further confirm the effect of SPM-mediated autophagy on regulation of M1/M2 polarization in KCs, ATG5-siRNA was used. Our data indicated that ATG5 knockdown significantly abolishes SPM-mediated autophagy in KCs. Furthermore, ATG5 knockdown restored genetic induction of M1 markers (*IL-1*β, *iNOS*) and M2 markers (*Arg-1, Mrc-1*), as well as the expression levels of STAT1 and STAT6, in TAA + SPM KCs. The protective role of SPM in TAA-induced acute liver injury was also abolished by knockdown of ATG5 in KCs *in vivo*. Our results are in agreement with other studies reporting that autophagy suppresses M1 polarization and promotes M2 polarization of macrophages, resulting in inhibition of inflammation.

In conclusion, this study demonstrates that SPM markedly attenuates TAA-induced acute liver injury. SPM-mediated KC autophagy, *via* upregulation of ATG5 expression, inhibits M1 polarization and promotes M2 polarization, which is responsible for attenuation of TAA-induced acute liver injury. Our findings suggest that SPM should be considered as a potent candidate for treating TAA-induced acute liver injury.

## Ethics Statement

All animals received humane care and all animal procedures met the relevant legal and ethical requirements according to a protocol (number NMU08-092) approved by the Institutional Animal Care and Use Committee of Nanjing Medical University.

## Author Contributions

FZ, LL, and HZ designed the research. SZ, JG, RL, SW, and QW performed the experiments. SZ and YD analyzed the data. SZ and HS wrote the manuscript.

## Conflict of Interest Statement

The authors declare that the research was conducted in the absence of any commercial or financial relationships that could be construed as a potential conflict of interest.
