# Supplementary material for: Spermine Alleviates Acute Liver Injury by Inhibiting Liver-Resident Macrophage Pro-Inflammatory Response Through ATG5-Dependent Autophagy
Source: Front Immunol. 2018 May 2;9:948. doi: 10.3389/fimmu.2018.00948 (PMC5940752; doi:10.3389/fimmu.2018.00948)
Supplement: Supplementary file 1 [file Presentation_1.PDF]

Supplementary Material

Spermine alleviates acute liver injury by inhibiting liver resident macrophage pro-inflammatory response through ATG5-dependent autophagy

Shun Zhou, Jian Gu, Rui Liu, Song Wei, Qi Wang, Yifan Dai, Hongbing Shen, Haoming Zhou*, Feng Zhang* and Ling Lu*

*** Correspondence:**

Feng Zhang

Email: zhangfeng1958@hotmail.com;

Ling Lu

Email: lvling@njmu.edu.cn;

Haoming Zhou

Email: hmzhou@njmu.edu.cn

Supplementary Figures

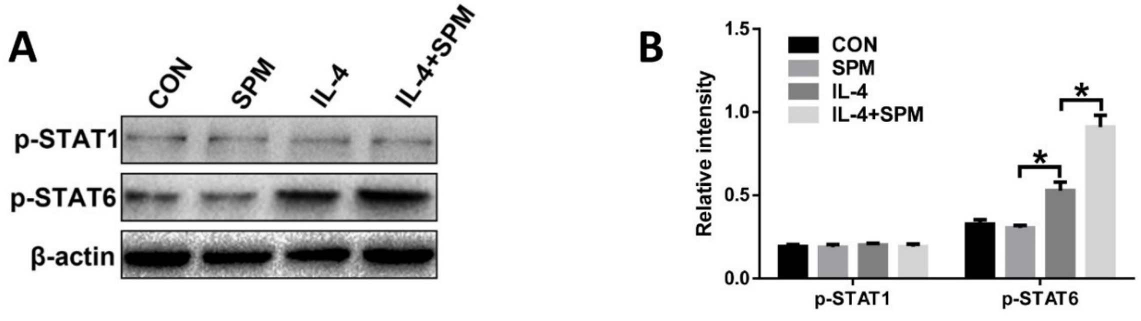

Supplementary Figure 1. SPM promotes KC M2 polarization in response to IL-4 stimulation in vitro. KCs isolated from mice were subjected to IL-4 stimulation in the presence or absence of SPM pretreatment as described in "Materials and Methods". Intracellular p-STAT1, p-STAT6 and β-actin protein levels were measured by Western blot. Representative of three experiments (**A**). Relative density ratios of target proteins in different experimental groups compared to the control group (CON) were calculated [(**B**), n=3/group].

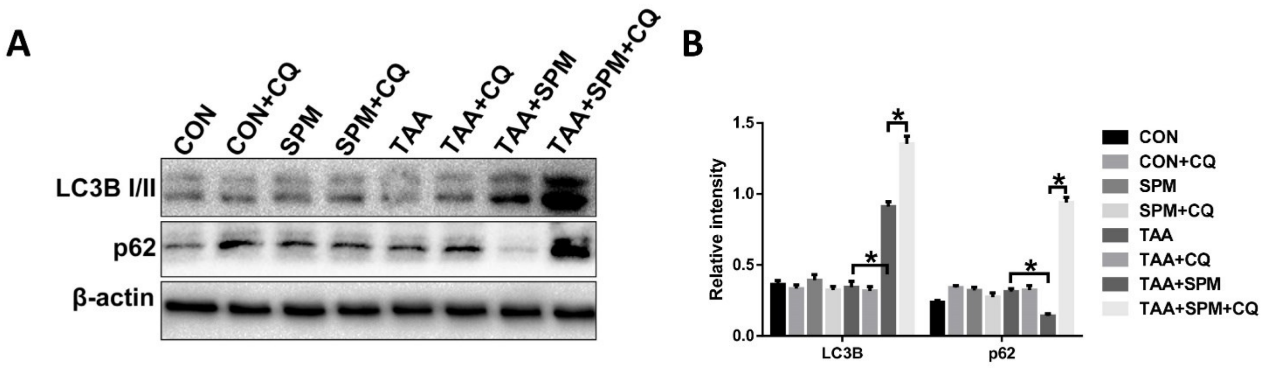

Supplementary Figure 2. SPM pretreatment promotes autophagic flux in KCs in response to TAA treatment. Mice were subjected to TAA administration in the presence or absence of SPM and CQ pretreatment as described in "Materials and Methods". KCs were isolated from different experimental groups, and intracellular LC3B, p62 and β-actin protein levels were measured by Western blot. Representative of three experiments (**A**). Relative density ratios of target proteins in different experimental groups compared to the control group (CON) were calculated [(**B**), n=3/group].

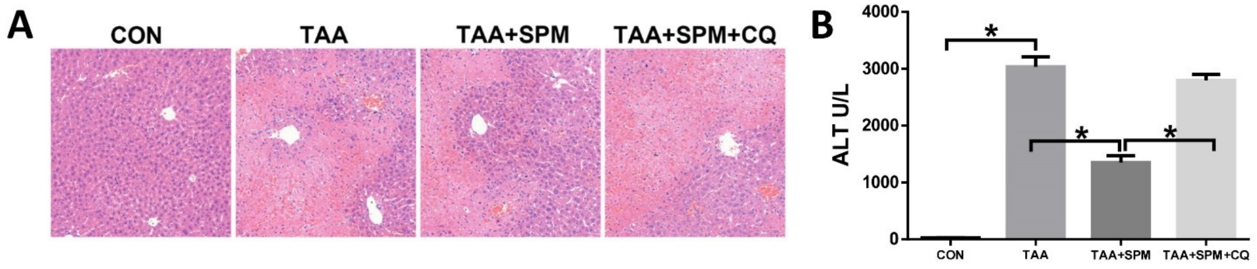

Supplementary Figure 3. Inhibition of autophagy abrogates the protective role of SPM in TAA-induced acute liver injury. Mice were subjected to TAA administration in the presence or absence of SPM and CQ pretreatment as described in “Materials and Methods”. Liver injury was evaluated in terms of liver histopathology [(**A**), representative of six mice/group] and serum ALT [(**B**), n=6/group]. (*p<0.05).

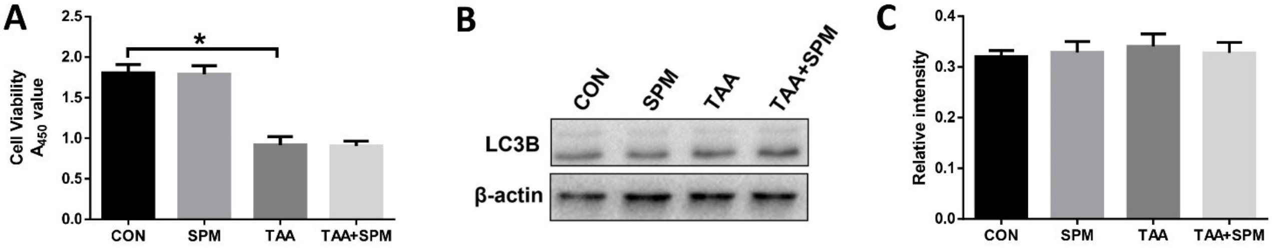

Supplementary Figure 4. SPM pretreatment has no significant effects on hepatocyte cell injury and autophagy activation post TAA treatment. Primary hepatocytes isolated from mice were subjected to TAA treatment in the presence or absence of SPM pretreatment as described in “Materials and Methods”. The viability of hepatocytes in different groups was determined by CCK-8 assay. Representative of three experiments (**A**). Intracellular LC3B and β-actin protein levels were measured by Western blot. Representative of three experiments (**B**). Relative density ratios of target proteins in different experimental groups compared to the control group (CON) were calculated [(**C**), n=3/group].
